# The Implications of Double Human Chorionic Gonadotropin (hCG) Trigger on a Pseudo-Empty Follicle Syndrome Patient Seeking Infertility Treatment Through Assisted Reproductive Technology (ART)

**DOI:** 10.7759/cureus.52783

**Published:** 2024-01-23

**Authors:** Ritesh Jadhav, Akash More, Shilpa Dutta, Namrata Anjankar, Jarul Shrivastava

**Affiliations:** 1 Clinical Embryology, Datta Meghe Institute of Higher Education and Research, Wardha, IND

**Keywords:** infertility treatment, empty follicle syndrome, oocyte retrieval, in vitro fertilization, double hcg trigger

## Abstract

In the field of assisted reproductive technology (ART), empty follicle syndrome (EFS) is a known condition in which no oocytes are found despite adequate follicular development, which leads to a troublesome situation for patients seeking infertility treatment. In this case study, an EFS patient seeking treatment for infertility at an in vitro fertilization (IVF) clinic was examined to determine the effects of employing a double human chorionic gonadotropin (hCG) trigger. The final oocyte maturation and retrieval are induced by using the double hCG trigger, which includes giving two doses of hCG. In this particular patient, the study looks at the results of follicular development, oocyte retrieval, fertilization rates, embryo quality, and pregnancy rates. The conclusion provides information on how well the double hCG trigger affects treatment outcomes for EFS patients. According to the results of this case study, the two-stage hCG trigger procedure is suggested to enhance the results of oocyte retrieval in the uncategorized EFS patient. In all cycles after the procedural change, the double trigger's application led to effective oocyte maturation and retrieval. The study also showed that the double hCG trigger procedure had no negative consequences on patient safety or ovarian response. There were also no signs of ovarian hyperstimulation syndrome (OHSS) or any other problems. Due to the higher likelihood of oocyte retrieval, the patient also reported better emotional health and less anxiety during subsequent treatment cycles. The positive result of this case study demonstrates the potential advantages of a double hCG trigger procedure in pseudo-EFS patients receiving IVF treatment. When handling EFS cases, this modified strategy may be used as a potential answer by infertility clinics. The effectiveness and safety of the double hCG trigger procedure still need to be confirmed by doing randomized controlled trials on larger populations in order to validate the result.

## Introduction

Infertility serves as a multifaceted and psychologically distressing phenomenon for individuals who, at present, desire to establish their own families via self-conception. It has been defined as when a couple is unable to conceive despite having regular physical contact for more than 12 calendar months [1]. The unprecedented increase in the implementation of assisted reproductive technology (ART) has been credited to a worldwide infertility prevalence ranging from 8% to 12% [2]. Within this niche, the phenomenon of empty follicle syndrome (EFS) contributes an additional level of complexity, leaving both patients and reproductive practitioners perplexed concerning its diagnosis and treatment. EFS has been defined as a condition in which it has been reported that despite having visual identification of a sufficient number of follicles via ultrasonography and the optimal response observed through controlled stimulation, no oocytes are retrieved during follicular screening [3]. It has been categorized into two types: genuine and false. The first type of condition can be described as a failure to recuperate oocytes from mature ovarian follicles following ovulation induction, notwithstanding normal follicular development and steroidogenesis, as well as appropriate beta-human chorionic gonadotropin (β-hCG) levels on the day of oocyte retrieval. The latter condition has been described as a failure to recover oocytes in the midst of low β-hCG due to an erroneous choice in transfer or the lesser bioavailability of choriogonadotropin [4]. False types generally occur due to the incorrect administration of hCG during controlled stimulation. However, there have been reported cases of 33% that have been discovered to be genuine [3]. The etiopathology of the genuine empty follicle syndrome (g-EFS) has been unclear despite several advances made by researchers in the study of the mechanism [5]. In our study, we had a patient for whom, despite observing optimal follicles during ultrasonography, no oocytes were retrieved during the ovum aspiration. Thereafter, she was diagnosed with EFS. However, it was not confirmed whether it was a case of false EFS or genuine EFS. Hence, we decided to employ a double hCG trigger mechanism to enhance the rate of oocyte retrieval in this patient. Thus, in a nutshell, this case report revolves around a patient diagnosed with EFS undergoing a double hCG trigger procedure to improve her chances of a successful clinical pregnancy.

## Case presentation

Patient's information

A 32-year-old female had a reported diagnosis of EFS. Oocyte retrieval was unsuccessful in both in vitro fertilization (IVF) cycles in the previous center, despite the existence of mature follicles. A double hCG trigger method was utilized in the third cycle to improve oocyte maturation and retrieval. Two doses of hCG were given in the double hCG trigger regimen, each at a 36.5-hour and 12.5-hour gap prior to ovum aspiration [6]. The goal of this technique was to increase the chance of oocyte retrieval in patients with EFS by offering additional stimulation for oocyte maturation. The patient came in with a history of two unsuccessful IVF cycles due to the non-retrieval of oocytes and was suggested for donor oocytes, in which the couple was disinterested. A double hCG trigger regimen was used in her third IVF cycle at our center. Using a gonadotropin-releasing hormone (GnRH) agonist technique, the patient underwent ovarian stimulation. Following follicular growth monitoring, the first hCG trigger (10,000 IU) was given 36.5 hours before ovum aspiration, when at least five follicles had grown to a diameter of 17 mm. A second hCG trigger (10,000 IU) was given about 12.5 hours later, prior to ovum aspiration. The patient underwent oocyte retrieval after the double hCG trigger procedure. Five oocytes from the right ovary and eight oocytes from the left ovary were successfully extracted during the process. The partner's sperm was subsequently used to perform intracytoplasmic sperm injection (ICSI) on these oocytes. Three of the 13 oocytes underwent fertilization, resulting in the development of three good-quality embryos. On day 5, two embryos were transplanted, and the third embryo was cryopreserved. The patient then had a successful singleton pregnancy after getting a positive pregnancy test.

Clinical findings

**Figure 1:**
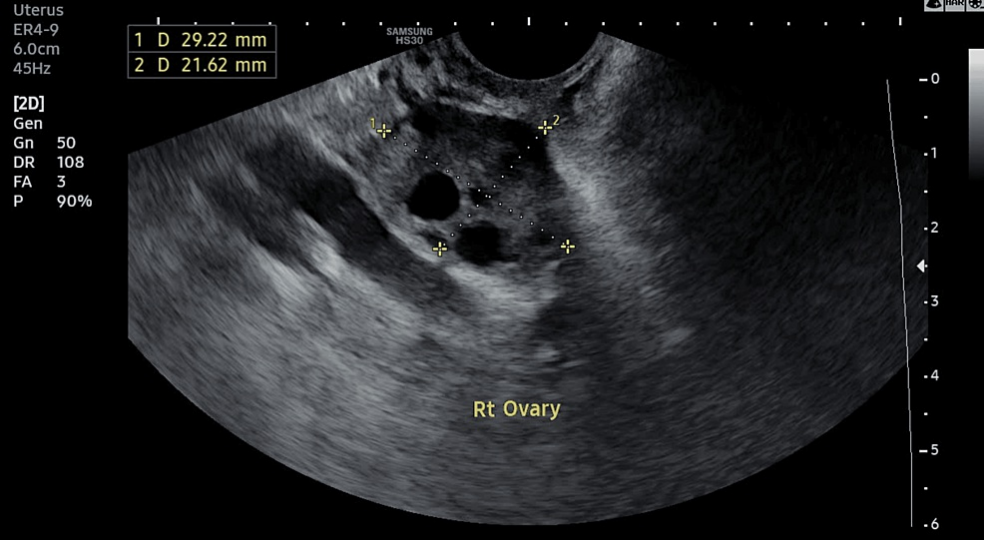
Right (Rt) ovary showing five follicles on day 2 via transvaginal sonography

**Figure 2:**
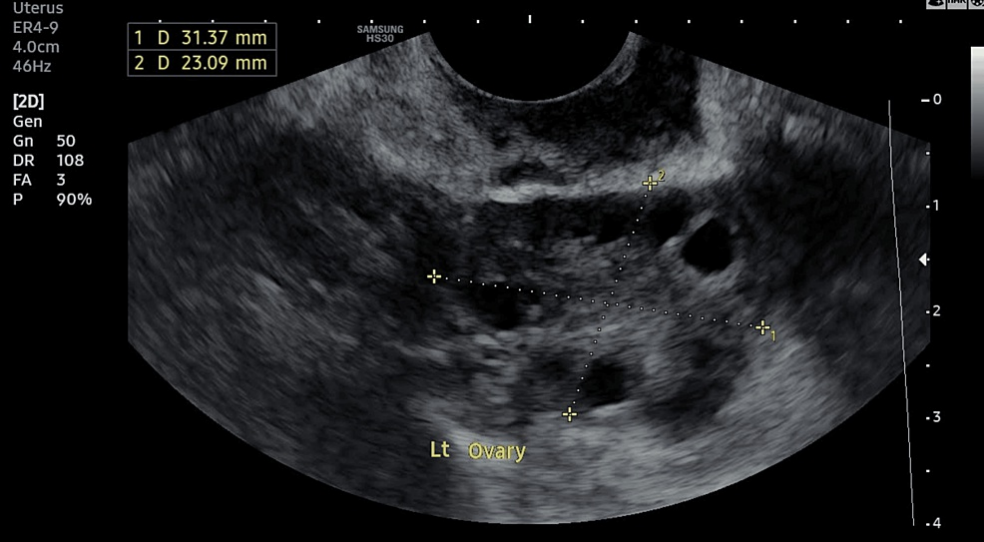
Left (Lt) ovary showing eight follicles on day 3 via transvaginal sonography

The positive pregnancy test served as an early indicator of the embryo's successful implantation into the uterine lining. This finding marked a crucial milestone in the fertility treatment process, suggesting a favorable environment for embryonic development. Overall, the clinical findings post transfer underscored the success of the assisted reproductive procedure, highlighting the effectiveness of the double hCG trigger in overcoming previous challenges associated with EFS. Figure 1 and Figure 2 represent the normal growth of follicles before the extraction of mature oocytes. Ultrasound reports of patient scans can be seen below.

Follow-up outcome

Compared to earlier attempts without the double hCG trigger, a significant number of mature oocytes were retrieved. The rates of fertilization were adequate, and a significant number of embryos were produced. Compared to earlier cycles, the embryo quality was significantly improved this time. The procedure to transfer the patient's embryos was successful. Fourteen days post embryo transfer, the patient was advised for a β-hCG test, which came out to be positive.

## Discussion

The intention behind using a two-step hCG trigger was to accelerate follicular development and encourage oocyte maturation. This strategy was intended to fix the problem that existed in earlier cycles, where the follicles did not have any oocytes [7]. The effective recovery of oocytes for fertilization is the main goal in patients with EFS [8]. The patient may have had more mature oocytes retrieved due to the double hCG trigger treatment. The second trigger's extra stimulus probably assisted in overcoming the earlier difficulties and increased oocyte retrieval rates. Oocyte retrieval and subsequent embryo development in this EFS patient significantly improved after using the double hCG stimulation [9]. To encourage oocyte maturation, increase the probability of successful fertilization, and promote embryonic development, two doses of hCG were given to patients.

The successful pregnancy in this example emphasizes the double hCG trigger protocol's potential advantages for EFS patients, particularly in terms of improved clinical results. There were more mature oocytes accessible, increasing the probability of fertilization [10]. Egg retrieval is performed before the onset of ovulation, which involves the rupture of a mature Graafian follicle and the release of the mature oocyte from the surface of the ovary [11]. It might have been possible that the double hCG trigger's improved oocyte retrieval rate had a good impact on fertilization rates, increasing the IVF procedure's overall success. The number of embryos recovered is a testament to the double hCG procedure's success. Due to improved oocyte quality and maturation, the use of a double hCG trigger may have produced better-quality embryos [12]. Embryos of higher quality have a higher chance of implantation and subsequent pregnancy. The two-step trigger approach most certainly contributed to the case's improved embryo retrieval rate and quality, which might have a favorable effect on the patient's chances of becoming pregnant [13].

The pursuit of effective treatments for infertility is centered around achieving a successful and healthy clinical pregnancy. One notable advancement in this realm is the utilization of the double human chorionic gonadotropin (hCG) trigger, a strategy designed to enhance various aspects of the reproductive process. This innovative approach has demonstrated significant improvements in several key stages of assisted reproductive technology (ART) [14]. The double hCG trigger's positive impact on follicular development is pivotal, fostering the growth and maturation of ovarian follicles, thereby increasing the likelihood of successful oocyte retrieval. This intervention also plays a crucial role in optimizing fertilization rates by ensuring a higher number of viable and mature oocytes. Consequently, the improved quality of embryos resulting from this technique contributes substantially to the overall success of assisted reproductive procedures. Moreover, the greater number of mature oocytes obtained through the double hCG trigger translates into enhanced prospects for successful implantation and the initiation of a continuing pregnancy [14]. The heightened embryonic quality further bolsters the chances of a sustained and healthy gestation. In essence, the double hCG trigger represents a multifaceted approach that positively influences various facets of the fertility treatment process, ultimately offering aspiring parents an increased probability of realizing their dream of parenthood [14]. However, this study was done on a single patient; hence, further studies are recommended especially with a larger sample population to validate the results.

## Conclusions

In this case study, the patient diagnosed with empty follicle syndrome responded favorably to the application of a double hCG trigger. The number of mature oocytes that could be increased with the use of the two-step hCG trigger procedure, which appears to enhance follicular development, and the higher retrieval rate of the oocytes available for fertilization and thereafter transfer may be a result of increased egg maturity and fertilization rates, which are suggested by the improved embryo quality. It is crucial to keep in mind, though, that every patient will experience their treatment differently and that certain patient-specific factors can affect success rates. This case report suggests that the patient might have been suffering from false EFS, due to which it responded positively during the double hCG trigger mechanism. Hence, the case study concludes that patients with uncategorized empty follicle syndrome seeking treatment for infertility may find the double HCG trigger treatment helpful.
